# A secreted AIP-Like peptide from *Helcococcus kunzii* inhibits the Agr quorum sensing system of *Staphylococcus aureus*

**DOI:** 10.1080/21505594.2026.2692776

**Published:** 2026-07-03

**Authors:** Riham Daher, Patrice Francois, Renaud Vincentelli, Annette C. Vergunst, Lucia Grenga, Cassandra Pouget, Julien Boyer, Nadia Gaïa, Jean-Philippe Lavigne, Catherine Dunyach-Remy

**Affiliations:** a VBIC University Montpellier INSERM U1047, Nîmes Cedex 2, France; b Genomic Research Laboratory, Service of Infectious Diseases, Medicine University of Geneva and Geneva University Hospitals, Geneva, Switzerland; c Architecture et Fonction des Macromolécules Biologiques, UMR7257 CNRS, Université Aix-Marseille, Marseille Cedex 9, France; d Laboratoire Innovations technologiques pour la Détection et le Diagnostic (Li2D), Service de Pharmacologie et Immunoanalyse (SPI), CEA, INRAE, Bagnols-sur-Cèze, France; e VBIC, INSERM U1047, Department of Microbiology and Hospital Hygiene, CHU Nîmes, University Montpellier, Nîmes Cedex 9, France

**Keywords:** *Staphylococcus aureus*, *Helcococcus kunzii*, Agr quorum sensing, biofilm formation, chronic wound infection, anti-virulence strategy, Adh2, zebrafish, proteomic, transcriptomic

## Abstract

*Staphylococcus aureus* is a major human pathogen whose virulence is tightly regulated by the Agr quorum sensing system. In this study, we investigated the impact of Adh2, a secreted protein from the commensal bacterium *Helcococcus kunzii*, on *S. aureus* physiology and pathogenicity. Adh2 shares structural similarity with native auto-inducing peptides (AIPs), including the conserved CDFIM motif characteristic of Agr group I. We hypothesized that Adh2 interferes with Agr signaling by competitively binding the AgrC receptor. Exposure to Adh2 significantly repressed *agrA* and its downstream α-hemolysin *hla*, while upregulating *spa*, a gene encoding a surface adhesin. Deletion of an Adh2 region encompassing the conserved CDFIM motif abolished this regulatory effect, indicating that this region is required for Adh2 activity. RNA-Seq analysis revealed global transcriptional reprogramming, with downregulation of virulence and metabolic genes. Proteomic profiling corroborated these findings, showing reduced abundance of proteins involved in metabolic pathways (e.g. carbohydrate, lipid, and nucleotide metabolism), consistent with a shift toward a low-energy, colonization-oriented state. Importantly, Adh2 did not impair *S. aureus* growth across a wide concentration range (0.01–10 g/L) but significantly enhanced biofilm formation. *In vivo*, Adh2 administration significantly improved survival in zebrafish embryos infected with *S. aureus*, validating its anti-virulence potential. Together, these findings demonstrate that Adh2 suppresses Agr signaling and virulence gene expression while promoting a persistent phenotype. By shifting *S. aureus* toward a metabolically reduced and less pathogenic state, Adh2 emerges as a promising candidate for therapeutic modulation of bacterial behavior, particularly in the context of chronic wound infections.

## Introduction

*Staphylococcus aureus is* a versatile opportunistic pathogen responsible for a wide range of infections, from superficial skin lesions to life-threatening conditions such as pneumonia, endocarditis, and sepsis [1]. A key determinant of its pathogenicity lies in its ability to tightly regulate the expression of virulence factors in response to environmental signals and population density [2]. This regulation is primarily orchestrated by the accessory gene regulator (Agr) quorum sensing system, which controls the expression of toxins, proteases, adhesins, and surface proteins [3].

Central to the Agr system are the autoinducing peptides (AIPs I–IV), derived from the AgrD precursor and processed by AgrB into a cyclic thiolactone peptide of 7–9 amino acids in length [4–8]. The mature AIP consists of a short N-terminal tail responsible for receptor activation and a hydrophobic thiolactone ring. In Agr group I strains, this ring contains the conserved CDFIM motif, which is essential for receptor recognition and docking [9]. Upon reaching a threshold concentration, AIP binds to the membrane-bound histidine kinase AgrC, triggering its autophosphorylation [10,11]. The phosphate group is then transferred to AgrA [12], which activates transcription from the P2 and P3 promoters, leading to RNAIII transcription [13]. RNAIII orchestrates a global shift in gene expression, repressing surface-associated genes while upregulating the expression of toxin genes, such as those encoding α-hemolysin (*hla*) and δ-hemolysin [13]. Sequence variations in AIPs define four Agr specificity groups (I–IV) [12], with cross-group AIP interactions leading to competitive inhibition of AgrC activation, a phenomenon known as “Agr interference” [12,14]. Given its central role in virulence regulation, the Agr system has emerged as a promising target for anti-virulence strategies aimed at disarming pathogens without exerting selective pressure for antibiotic resistance [15–17]. Among these strategies, quorum sensing interference using AIP mimics or antagonists is gaining attention [14,18]. Molecules structurally similar to native AIPs can potentially bind AgrC without activating it, thereby acting as competitive inhibitors [19].

In this study, we investigated a candidate peptide-based quorum-sensing inhibitor derived from *Helcococcus kunzii* H13, a Gram-positive commensal bacterium previously shown to attenuate the virulence of *S. aureus* NSA739, a clinical isolate from grade 3 diabetic foot infections in a *Caenorhabditis elegans* infection model [20]. Genomic and proteomic analyses identified a secreted protein, Adh2 (Alcohol dehydrogenase 2, GUI37_RS02525), containing the conserved AIP-I motif “CDFIM” [9,21,22]. Based on this sequence similarity and the presence of this conserved motif CDFIM, which is known to contribute to the hydrophobic cyclic structure required for AgrC receptor binding, we hypothesized that Adh2 could act as a functional mimic, interfering with Agr signaling by competing for AgrC binding [23].

To test this hypothesis, we evaluated the impact of Adh2 on the expression of key Agr-regulated genes (*agrA*, *hla*, *spa*) using quantitative PCR. We further performed transcriptomic and proteomic analyses to characterize global changes in *S. aureus* gene and protein expression following Adh2 exposure. Additionally, we evaluated the effects of Adh2 on bacterial growth and biofilm formation *in vitro* and validated its anti-virulence potential in a zebrafish embryo infection model.

Together, this work reveals a novel mechanism by which a commensal bacterium modulates *S. aureus* virulence and identifies Adh2 as a promising anti-virulence agent targeting the Agr system.

## Materials and methods

### Bacterial strains and culture conditions

The bacterial strain used in this study was *S. aureus* NSA739 (Agr type I), a virulent clinical isolate obtained from a grade 3 diabetic foot infection. All experiments were conducted using Chronic wound medium (CWM), a proprietary culture medium developed and patented by our research team to mimic the physiological conditions of chronic wounds [24]. The chronic wound medium (CWM) was formulated as described previously, consisting of 79.5% Bolton broth (Sigma-Aldrich, ref. 67,454-500 G), supplemented with 20% heat-inactivated human serum (EFS) and 0.5% hemolyzed human blood (EFS) [24]. For zebrafish experiments, a GFP-expressing derivative of NSA739 (NSA739-GFP) was used to facilitate visualization of host–pathogen interactions. For all assays, NSA739 cultures were initiated by diluting overnight precultures to an initial optical density at 600 nm (OD_6__00_) of 0.1, followed by incubation at 37°C with shaking (200 rpm) for 2.5–3 h. This growth period allowed the bacteria to reach the late exponential phase (OD_6__00_ between 0.6 and 0.7), corresponding to activation of the Agr quorum sensing system. At this stage, Adh2 protein or control treatments were applied according to the experimental design.

### Protein production and purification

The coding sequences of wild-type Adh2 (44.3 kDa) and a mutant variant, mch_mut_Adh2 (65.7 kDa), in which the AIP-like CDFIM motif was deleted, were cloned into a modified pET28 vector (kanamycin resistance) containing an N-terminal His_6_ tag and a TEV protease cleavage site (Twist Bioscience, San Francisco CA, USA). Due to technical limitations encountered during the expression and purification of the mutant Adh2 protein alone, a fusion with mCherry was introduced to improve protein stability and yield. A corresponding mCherry-Adh2 construct was generated as a control to account for any potential effects of the fusion tag. The mch_mut_Adh2 construct was designed to remove not only the conserved CDFIM motif but also the surrounding amino acid region (~60 residues) to ensure complete disruption of the putative AIP-like functional domain and prevent potential structural compensation or residual activity. This design aimed to abolish the functional mimicry of the native AIP motif while maintaining overall protein expression and stability. To achieve this, the clone was introduced in the modified pET28 vector with the mCherry added between the His and TEV site. A third construct, mcherry_Adh2 (mch_Adh2) (72 kDa), was produced as a control for the mCherry fusion. The full-length protein sequences of Adh2, the mutant variant mch_mut_Adh2, and mch_Adh2 are provided below.

The full-length amino acid sequence of Adh2 (with initial glycine and stop codon) is as follows: GEFVLPAINKFGKDSYQEISNTLLKENKKKGLIITTKDIYELGLLKDILNILEENQIQYEMYTGVVPNPTINNVEDSSKLFLDNKCDFIMGVGGGSANDCAKAVGILVSNGGNIKEYVGFNKSRHKSPFLICVNTTAGTASEISRAFLINDEENKEKLIFKDINALPDLSINNPEIMFGLPKKITAQTGMDALTHAIESYISTGAYTLTKEFSLSAIKLIFNSLEKVIDEPKNENLRNEMIYAQFLAGMAFCNAGLGLVHAMSHQLSALYNLPHGLSNAILLPEVMRLNKKTSTKGFAEIYQNIFLDSFEDDEKSADLLIERVENLSDKVGTKVKLSDLGVLEEDIDKLIDMTFKDGNLPRNPYQPTKEDVKNIFKKVM.

The full amino acid sequence of the His_6_-mcherry-mut_Adh2 fusion (with initial glycine and stop codon) is:

MGSSHHHHHHSSRVSKGEEDNMAIIKEFMRFKVHMEGSVNGHEFEIEGEGEGRPYEGTQTAKLKVTKGGPLPFAWDILSPQFMYGSKAYVKHPADIPDYLKLSFPEGFKWERVMNFEDGGVVTVTQDSSLQDGEFIYKVKLRGTNFPSDGPVMQKKTMGWEASSERMYPEDGALKGEIKQRLKLKDGGHYDAEVKTTYKAKKPVQLPGAYNVNIKLDITSHNEDYTIVEQYERAEGRHSTGGMDELYKPQPKLRSSGENLYFQGEFVLPAINKFGKDSYQEISNTLLKENKKKGLIITTKDIYELGLLKDILNILEENQIQYENKSRHKSPFLICVNTTAGTASEISRAFLINDEENKEKLIFKDINALPDLSINNPEIMFGLPKKITAQTGMDALTHAIESYISTGAYTLTKEFSLSAIKLIFNSLEKVIDEPKNENLRNEMIYAQFLAGMAFCNAGLGLVHAMSHQLSALYNLPHGLSNAILLPEVMRLNKKTSTKGFAEIYQNIFLDSFEDDEKSADLLIERVENLSDKVGTKVKLSDLGVLEEDIDKLIDMTFKDGNLPRNPYQPTKEDVKNIFKKVM.

The complete amino acid sequence of His_6_-mcherry_Adh2 (with initial glycine and stop codon) is: MGSSHHHHHHSSRVSKGEEDNMAIIKEFMRFKVHMEGSVNGHEFEIEGEGEGRPYEGTQTAKLKVTKGGPLPFAWDILSPQFMYGSKAYVKHPADIPDYLKLSFPEGFKWERVMNFEDGGVVTVTQDSSLQDGEFIYKVKLRGTNFPSDGPVMQKKTMGWEASSERMYPEDGALKGEIKQRLKLKDGGHYDAEVKTTYKAKKPVQLPGAYNVNIKLDITSHNEDYTIVEQYERAEGRHSTGGMDELYKPQPKLRSSGENLYFQGEFVLPAINKFGKDSYQEISNTLLKENKKKGLIITTKDIYELGLLKDILNILEENQIQYEMYTGVVPNPTINNVEDSSKLFLDNKCDFIMGVGGGSANDCAKAVGILVSNGGNIKEYVGFNKSRHKSPFLICVNTTAGTASEISRAFLINDEENKEKLIFKDINALPDLSINNPEIMFGLPKKITAQTGMDALTHAIESYISTGAYTLTKEFSLSAIKLIFNSLEKVIDEPKNENLRNEMIYAQFLAGMAFCNAGLGLVHAMSHQLSALYNLPHGLSNAILLPEVMRLNKKTSTKGFAEIYQNIFLDSFEDDEKSADLLIERVENLSDKVGTKVKLSDLGVLEEDIDKLIDMTFKDGNLPRNPYQPTKEDVKNIFKKVM**. Sequence alignments between wild-type Adh2 and mch_mut_Adh2 (Figure S4), and between mCherry-Adh2 and mCherry-mutant Adh2 (Figure S5), are provided to clearly illustrate the deletion of the CDFIM-containing region and confirm the structural integrity of the fusion constructs.

Protein expression and purification followed a protocol adapted from [25] with detailed procedures described in the following section. Constructs were expressed in *Escherichia coli* BL21 (DE3) using an auto-induction medium (NZytech, Lisboa, Portugal). Cultures were grown at 37°C for 4 h, then incubated overnight at 17°C to enhance protein solubility. Cells were harvested by centrifugation and lysed in buffer A (50 mM Tris-HCl pH 8.0, 300 mM NaCl, 10 mM imidazole) supplemented with lysozyme and DNase I. Lysis was performed by sonication, and lysates were clarified by centrifugation [25]. Soluble His-tagged proteins were purified under native conditions using nickel-affinity chromatography (HisTrap FF crude 5 mL column on AKTA Xpress; Cytiva, Saint-Germain-en-Laye, France) and eluted with buffer A containing 250 mM imidazole. Protein purity was assessed by SDS-PAGE (Figure S1) using a 4–20% Mini-PROTEAN® TGX Stain-Free™ Gel (Bio-Rad, ref. #4568093EDU) and an Unstained Protein Molecular Weight Marker (14.4–116 kDa; Euromedex, ref. 06 U-0511) as a molecular weight standard. Eluted proteins were pooled, concentrated, and buffer-exchanged into phosphate-buffered saline (PBS, pH 7.4). Final concentrations, measured by absorbance at 280 nm, were 10 g/L for Adh2, 1.5 g/L for mch_mut_Adh2, and 11 g/L for mch_Adh2. Proteins were aliquoted and stored at −80°C until use.

### Quantitative RT-PCR analysis

The expression levels of *agrA*, *hla*, and *spa* in *S. aureus* NSA739 were assessed by quantitative real-time PCR (qPCR), following a protocol adapted from Doumith *et al.* [26], with detailed procedures described in the following section. Total RNA was extracted from bacterial cultures grown in CWM, either untreated or treated with Adh2 (0.01 g/L or 10 g/L), or with the Adh2 mutant lacking the CDFIM motif (mut_Adh2), using the RNeasy® Mini Kit (Qiagen, Courtaboeuf, France) according to the manufacturer’s instructions. To eliminate residual genomic DNA, samples were treated with RNase-free DNase I (Qiagen) at 37°C for 30 min. RNA quantity and purity were verified using a NanoDrop™ 2000 spectrophotometer (Fisher Scientific, Pittsburgh, PA, USA). Subsequently, 1 µg of total RNA was reverse transcribed into cDNA using the iScript™ Select cDNA Synthesis Kit (Bio-Rad). Quantitative PCR was carried out using the LightCycler® 480 system (Roche) and the LightCycler® RNA Master SYBR Green I kit (Roche Applied Science, Meylan, France). Reactions were performed in triplicate, and amplification specificity was confirmed by melting curve analysis. Gene expression levels were normalized to the housekeeping gene *gyrB*, used as an internal reference for *S. aureus*. Relative quantification was calculated using the 2^−ΔΔCt method, where ΔCt = Ct target gene−Ct housekeeping gene *gyrB*, and ΔΔCt represents the difference between treated and control conditions (±Adh2 or mut_Adh2). Expression was evaluated at 1 h (to detect early transcriptional responses) and 24 h (to assess persistence over time). The primers used in this study were as follows: *agrA*-F: 5′-CAA AGA GAA AAC ATG GTT ACC ATT ATT AA-3′, *agrA*-R: 5′-CTC AAG CAC CTC ATA AGG ATT ATC AG-3′, *hla*-F: 5′-TCC AGT GCA ATT GGT AGT CA-3′, *hla*-R: 5′-GGC TCT ATG AAA GCA GCA GA-3′, *spa*-F: 5′-TAT GCC TAA CTT AAA TGC TG-3′, *spa*-R: 5′-TTG GAG CTT GAG AGT CAT TA-3′, *gyrB*-F: 5′-GAT GAA GCG TTT ATG CTC GC-3′, *gyrB*-R: 5′-AGT GCG TTG TCC AAT GTT GC-3′.

### RNA-Seq sample preparation and transcriptomic analysis

Transcriptomic profiling was performed on *S. aureus* NSA739 treated with Adh2 or PBS. Overnight cultures were diluted into fresh CWM to an initial OD_600_ of 0.1 and grown at 37°C for 2.5 to 3 h until reaching an OD_600_ between 0.6 and 0.7. At this point, Adh2 was added to the experimental samples at a final concentration of 0.01 g/L for 1 h. PBS served as control. Three biological replicates were prepared per condition. Total RNA was extracted using the RNeasy Plus Mini Kit (Qiagen), quantified, and 1 µg per sample was subjected to ribosomal RNA depletion using the Ribo-Zero Bacterial kit (Illumina, San Diego CA, USA). Libraries were prepared using the TruSeq Total RNA Stranded kit (Illumina), quantified with Qubit, and quality-checked on a TapeStation system (Agilent Technologies, Santa Clara CA, USA). Equimolar pooling was performed, and the final library pool was loaded at 2 nM for clustering. Libraries were sequenced on an Illumina HiSeq 4000 platform using oriented single-end, 50-bp reads, yielding at least 50 million mapped reads per sample (iGE3 Genomics Platform, University of Geneva, Switzerland). Transcriptomic data analysis was performed using DESeq2 and the Python 3 environment on Google Colab [27]. Statistical testing was conducted in R v3.2.3 using the EdgeR package [28,29]. Genes with <1 count per million in ≥2 replicates were excluded. Remaining genes were normalized using the trimmed mean of *M* values (TMM). Read counts were modeled with a negative binomial distribution, and pairwise comparisons between groups were performed using the exact test to identify differentially expressed genes (DEGs) [29,30]. *p*-values were adjusted using the false discovery rate (FDR = 0.05) correction.

### Proteome and exoproteome analyses of S. aureus exposed to Adh2

Overnight cultures of *S. aureus* NSA739 were grown in CWM under standard conditions for 18 h. Bacterial suspensions were adjusted to an OD_600_ of 0.1 and inoculated into 6 mL volumes in 25 cm^3^ flasks containing solidified CWM. Cultures were incubated at 37°C for 24 h under constant agitation (50 rpm). After incubation, cultures were diluted to an OD_600_ of 0.1 in fresh liquid CWM and grown for 2.5 to 3 h to reach late exponential phase (OD_6__00_ = 0.6–0.7). At this point, Adh2 was added to the test group at 0.01 g/L; PBS served as control. All conditions were performed in biological triplicate. After 1 hour of exposure to Adh2 or PBS, cultures were harvested. Proteome extraction followed previously described protocols [22] with details provided below. Cells were centrifuged (4,000 rpm, 10 min at 4°C), washed once with 1 × PBS, and the resulting pellets were centrifuged again (10,000 rpm, 3 min). Pellets were resuspended in LDS 1× buffer (NuPAGE, ThermoFisher) with 5% β-mercaptoethanol (Sigma-Aldrich, Saint-Quentin Fallavier) and stored at −20°C until further processing. For exoproteome extraction, culture supernatants were collected immediately after centrifugation and filtered through 0.22 μm syringe filters (VWR, Rosny-sous-Bois, France) to remove residual cells. Proteins in the filtrates were precipitated using chloroform-methanol extraction [31]. The resulting pellets were air-dried, resuspended in LDS 1× buffer with 5% β-mercaptoethanol, and stored at −20°C. Protein extracts were enzymatically digested into tryptic peptides and analyzed using a nano-LC system (UltiMate 3000, ThermoFisher Scientific) coupled to a high-resolution Exploris 480 mass spectrometer (ThermoFisher Scientific) [22]. Peptides were desalted on a PepMap 100 C18 μ-precolumn (5 μm, 100 Å, 300 μm i.d. ×5 mm), separated on a PepMap 100 C18 analytical column (3 μm, 100 Å, 75 μm i.d. ×50 cm) with a 95-min gradient at 0.2 μL/min (90 min from 5% to 25% of solvent B (100% acetonitrile, 0.1% formic acid), followed by 5 min increasing from 25% to 40%. Solvent A consisted of 0.1% formic acid in water. MS data acquisition was performed in Top 20 mode (350 to 1500 m/z), with dynamic exclusion (10 s) and selection of +2 or +3 charged precursors (2.0 m/z isolation window). Raw data were processed using Mascot Daemon v2.6.0 (Matrix Sciences, Chicago, IL, USA) against a custom protein database including *S. aureus* NSA739 (biosample SAMN39991533), *Homo sapiens*, and the recombinant Adh2 protein. Each condition (Adh2 or PBS) was performed in biological triplicate, for both proteome and exoproteome samples. Differential protein expression was considered significant with fold change ≥1.5 or ≤–1.5, and a *p* < 0.05. Functional analysis for the identified proteins was obtained using the BLAST Koala tool (https://www.kegg.jp/blastkoala/). The corresponding KEGG pathways and functional categories were then assigned using the KEGG Mapper–Reconstruct Pathway tool (https://www.genome.jp/kegg/mapper/reconstruct.html). Signal peptide prediction was done using SignalP 6.0 (//services.healthtech.dtu.dk/services/SignalP-6.0/).

### Growth curves analysis

*S. aureus* NSA739 cultures were prepared in CWM at OD_6__00_ of 0.1. Adh2 was added to the bacterial solution at varying concentrations; PBS served as control. Two hundred μL of each culture were dispensed into 96-well flat-bottom microplates (Costar Corning, New York, NY, USA) and incubated at 37°C in a Tecan Infinite F200 microplate reader with continuous shaking at 200 rpm. OD_6__00_ measurements were recorded hourly. Growth kinetics were modeled using the Gompertz growth model. Three independent biological experiments were performed.

### Biofilm biomass quantification

NSA739 cultures were grown in CWM to OD_6__00_ of 0.1 and incubated for 3 h at 37°C with shaking to reach late exponential phase (OD_6__00_ = 0.6–0.7) and then treated with Adh2 at 0.01 g/L or 10 g/L. PBS served as control. Aliquots (500 μL) were transferred to sterile 48-well flat-bottom plates (Costar Corning) and incubated statically at 37°C for 24 h. After incubation, wells were gently rinsed with PBS to remove planktonic cells, and biofilm-associated cells were resuspended in 500 μL of PBS. Biofilms were disrupted by sonication (10 min, 40 kHz) and viable cell density was quantified using the QUANTOM™ Cell Counter (Logos Biosystems, Villeneuve d’Asq, France), expressed as CFU/mL. Three biological replicates were performed. Statistical significance was assessed using unpaired *t*-tests.

### Zebrafish infection Model

Infection assays were performed using zebrafish (*Danio rerio*) embryos as previously described [32], full experimental details and specific modifications are provided in the following section. NSA739-GFP, carrying the plasmid pHOM-GFP, was grown overnight at 37°C in CWM with tetracycline for plasmid maintenance. Cultures were diluted in fresh CWM and grown to an exponential phase. Bacterial suspensions were washed twice with PBS and then adjusted to a final concentration of approximately 2,500 colony-forming units (CFU) per nL. At 30-h post-fertilization (hpf), zebrafish embryos from Tg(*mpx: Gal4-VP16*^i222^/*UAS-E1b:nfsBmCherry*^i149^) [33–35], expressing mCherry in neutrophils, were manually dechorionated and anesthetized in E3 medium with 0.02% buffered tricaine methanesulfonate (MS222). Groups of embryos (on average *n* = 50 per condition) were microinjected into the caudal vein with 1 nL of bacterial suspension. In treatment groups, Adh2 protein (10 g/L) was either co-injected or administered intravenously at 2 h post-infection (hpi). Control embryos received bacteria alone or bacteria + PBS at 2 hpi. Inoculum size was verified by plating PBS droplets from injection needles on LB agar containing tetracycline at regular intervals (three plates per needle). Embryos were maintained at 28°C in E3 medium. Mortality was evaluated at 24, 48, and 72 hpi by absence of heartbeat (on average *n* = 35 per condition). Bacterial load was quantified in 5 individual embryos per condition at 24 and 48 hpi (Figure S3). Three independent biological replicates were performed. Survival data were analyzed using Kaplan–Meier curves, and statistical significance was determined using the Log-rank (Mantel–Cox) test. CFU data was visualized as violin plots with scattered data points. Normality was confirmed by D’Agostino & Pearson test. Statistical significance was determined using one-way Anova with Sidaks’ multiple comparison test.

## Results

### Deletion of an Adh2 region containing the conserved CDFIM motif abolishes Agr system Inhibition

To assess the impact of Adh2 on the Agr quorum sensing system and associated virulence gene expression in *S. aureus*, we quantified the expression of three key markers *agrA*, *hla* and *spa* using qPCR. Adh2 was added to bacterial cultures at two concentrations: a low dose (0.01 g/L), and a high dose (10 g/L), at OD_600_ = 0.7.

**Figure 1.**
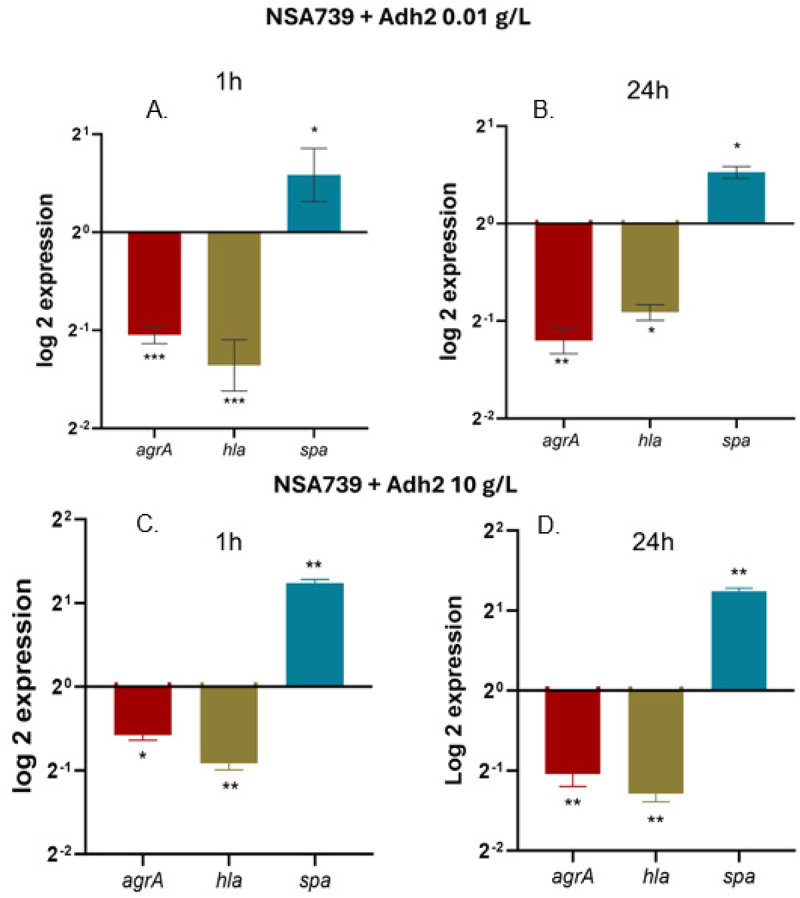
Adh2 represses expression of agrA and hla and upregulates spa in *Staphylococcus aureus* NSA739 at both tested concentrations. Gene expression was measured by RT-qPCR and expressed as log_2_ Fold change relative to untreated controls (NSA739 without Adh2), normalized to the housekeeping gene gyrB. Expression levels for the genes agrA, hla, and spa are shown after 1 h (A) and 24 h (B) exposure to Adh2 at 0.01 g/L, and after 1 h (C) and 24 h (D) exposure to Adh2 at 10 g/L. Bars represent the logarithmic mean ± standard deviation from three independent experiments, each performed in duplicate. Statistical significance was determined using unpaired t-test (* *p* < 0.05; ** *p* < 0.01; *** *p* < 0.001).

At 0.01 g/L, Adh2 significantly downregulated *agrA* and *hla* expression at 1-h post-exposure (Figure 1(A)). This repression persisted at 24 h (Figure 1(B)), indicating a sustained inhibitory effect even at low concentrations. In contrast, *spa* was upregulated at both timepoints, consistent with *agrA* inhibition (Figure 1(A,B)).

Exposure to the higher Adh2 concentration (10 g/L) yielded similar regulatory trends: repression of *agrA* and *hla*, and upregulation of *spa* at 1 and 24 h (Figure 1(C,D)). Notably, *spa* expression was more pronounced at 10 g/L compared to 0.01 g/L, suggesting a concentration-dependent effect. These findings confirm that Adh2 modulates Agr-regulated gene expression, repressing virulence factors (*agrA*, *hla*) while enhancing adhesion expression (*spa*).

**Figure 2.**
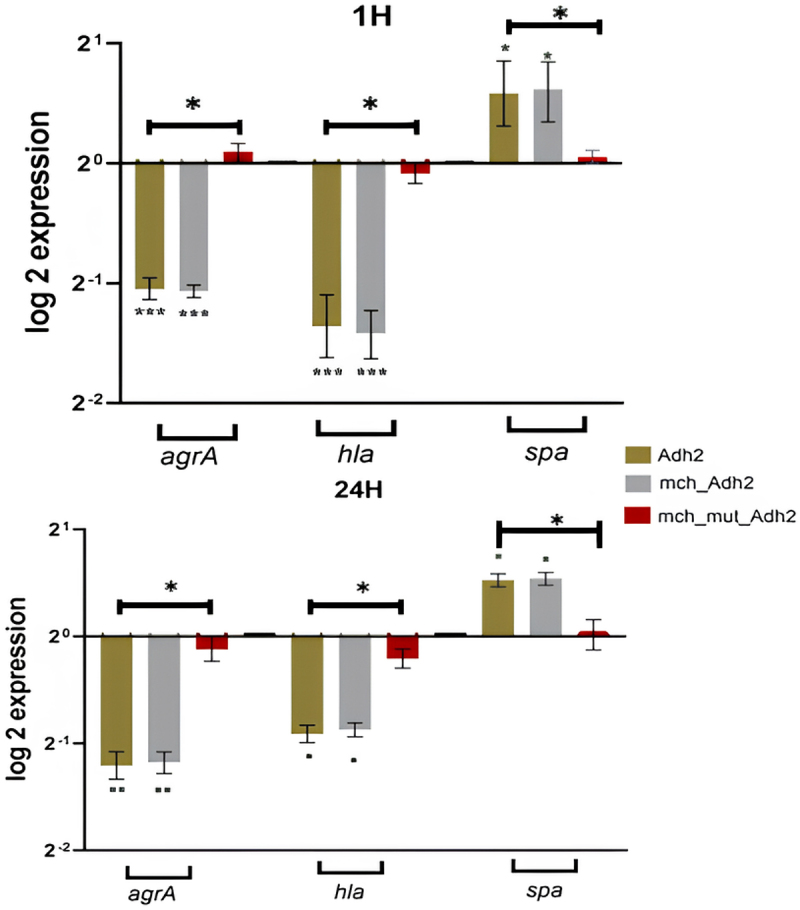
Deletion of an Adh2 region encompassing the conserved CDFIM motif abolishes Agr-modulatory activity in *Staphylococcus aureus*. Expression of agrA and hla was significantly repressed by Adh2 (0.01 g/L) and mch_Adh2 (0.01 g/L), while the mutant mch_mut_Adh2 (0.01 g/L) carrying a deletion of a larger region containing the conserved CDFIM motif, did not significantly affect gene expression. Data are expressed as log_2_ Fold change relative to untreated controls (PBS), normalized to the housekeeping gene gyrB. Bars represent mean ± standard deviation from three independent experiments, each performed in duplicate. Statistical comparisons were performed using unpaired t-test. Asterisks above individual bars indicate comparisons between each treatment (Adh2, mch_Adh2, or mch_mut_Adh2) and the PBS control. Horizontal lines with asterisks indicate comparisons between Adh2 and mch_mut_Adh2. (* *p* < 0.05; ** *p* < 0.01; *** *p* < 0.001).

To determine whether this inhibitory effect was dependent on the AIP-like motif in Adh2, we generated a mutant lacking a larger Adh2 region encompassing the conserved CDFIM pentapeptide (AIP-I motif). The mutant protein (mch_mut_Adh2) was produced and purified as an mCherry fusion. Wild-type Adh2 fused to mCherry (mch_Adh2) was used as a control to account for potential structural interference caused by the fusion. As shown in Figure 2, both Adh2 and mch_Adh2 repressed *agrA* and *hla* and upregulated *spa* at 1 and 24 h. In contrast, mch_mut_Adh2 did not significantly alter the expression of any of the three genes, with levels comparable to untreated controls. Statistical analysis confirmed significant differences between wild-type and mutant treatments for all three genes at both timepoints (*p* < 0.05). These results indicate that the deleted region containing the conserved CDFIM motif is required for the Agr-modulatory activity of Adh2.

### Impact of Adh2 on the transcriptomic profile of S. aureus

RNA-seq analysis was performed on triplicate samples treated with Adh2 (0.01 g/L) or PBS (Table S1). A total of 229 genes were differentially expressed between Adh2-treated and control conditions (Figure S2A), with 131 significantly downregulated and 98 upregulated. The remaining 2,051 genes showed no significant change. A volcano plot (Figure S2B) illustrates the magnitude and significance of these changes.

Among the DEGs, 48 genes encoded hypothetical proteins with unknown functions (Table S2), representing potential novel regulatory or structural targets.

**Figure 3.**
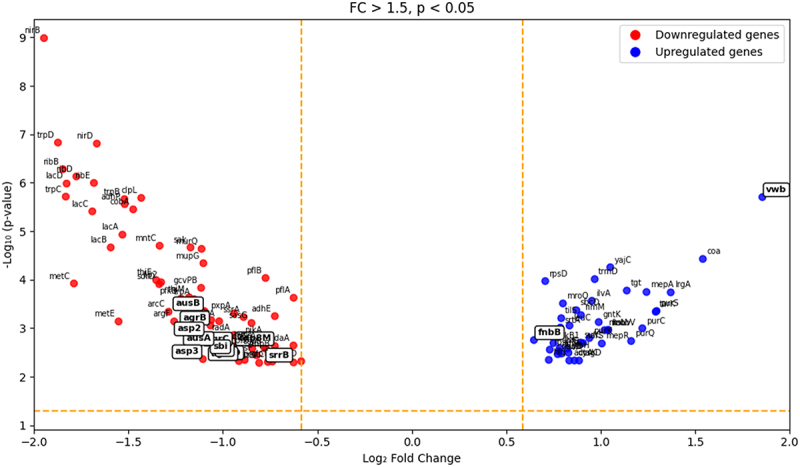
Volcano plot of annotated differentially expressed genes (DEGs) in *S. aureus* following Adh2 exposure (0.01 g/L). Each point represents a gene; significantly downregulated genes are shown in red, upregulated genes in blue. Volcano plot was generated using Python 3 scripts in the Google Colab environment. DEG was considered significant with Fold change ≥ 1.5 or ≤–1.5, and a *p* < 0.05. Genes discussed in the main text are highlighted with boxes.

All DEGs are listed in Table S3, including annotated genes (with known protein products) and unannotated genes (with identified protein functions but lacking gene names). For clarity, only annotated genes are shown in the volcano plot (Figure 3).

Transcriptomic analysis revealed strong repression of the Agr quorum sensing system (Table S3), with *agrA* (log2FC = −0.92, *p* = 0.004), *agrB* (log2FC = −1.10, *p* = 0.0008), *agrC* (log2FC = −0.95, *p* = 0.002), and *agrD* (log2FC = −0.88, *p* = 0.004), confirming qPCR findings. Beyond Agr, Adh2 also affected other regulatory pathways. The two-component system *srrAB*, involved in oxygen sensing, oxidative stress response, and anaerobic metabolism, was repressed (*srrB*, log2FC = −0.63, *p* = 0.005). Additional downregulated genes included *cap8M* (capsule biosynthesis; log2FC = −0.72, *p* = 0.002), *atl* (autolysin; log2FC = −0.94, *p* = 0.0039), and the aureusimine biosynthesis genes *ausA* (log2FC = −1.04, *p* = 0.002) and *ausB* (log2FC = −1.10, *p* = 0.0004). The immunoglobulin-binding protein gene *sbi* (log2FC = −0.95, *p* = 0.0034) and accessory Sec secretion system genes *asp2* (log2FC = −1.10, *p* = 0.001) and *asp3* (log2FC = −1.11, *p* = 0.004) were also significantly repressed. The main differentially expressed genes discussed above are highlighted in Figure 3.

**Figure 4.**
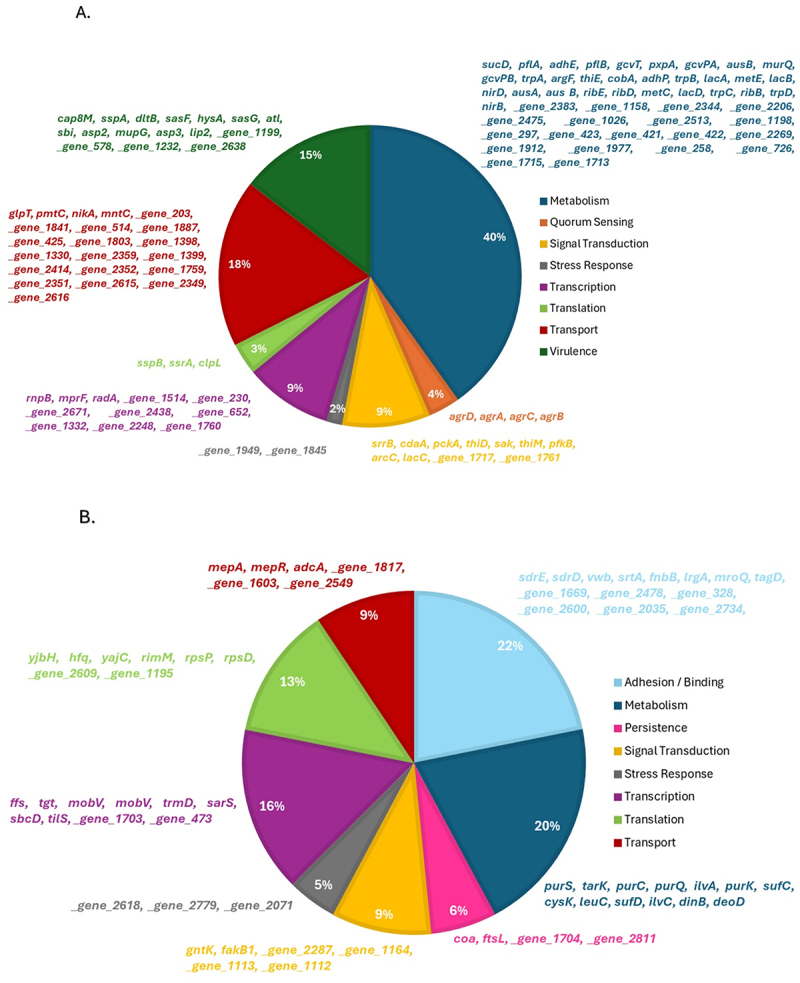
Functional classification of differentially expressed genes *(DEGs)* down-regulated (A) and up-regulated (B) in *S. aureus* following Adh2 exposure (0.01 g/L) according to their functional classes. Functional categories were assigned using UniProt and KEGG annotations. Each color indicates a specific functional class. The gene names associated with each class are indicated around the chart, color-coded to match their respective functional categories.

Functional classification using KEGG annotations showed that most downregulated genes were involved in metabolism, transport and virulence (Figure 4(A)). In contrast, up-regulated genes were enriched in bacterial adhesion and host protein binding functions, including *fnbB* and *vwb*, as well as transcriptional and translational regulation (Figure 4(B)).

In summary, Adh2 exposure induces a global transcriptional shift in *S. aureus*, characterized by repression of virulence and metabolic genes, and activation of adhesion and stress-response pathways.

### Proteomic profiling of S. aureus exposed to Adh2

To complement transcriptomic data, proteomic and exoproteomic analyses were performed on *S. aureus* NSA739 following 1-h exposure to Adh2 at 0.01 g/L, in triplicate, alongside PBS-treated controls. A total of 1,641 proteins were identified (Table S4), representing approximately 61% of the predicted proteome of NSA739. Differential analysis revealed 79 proteins in the cellular proteome (Table S6) and 17 in the exoproteome (Table S7) with significant changes (*p* < 0.05).

**Figure 5.**
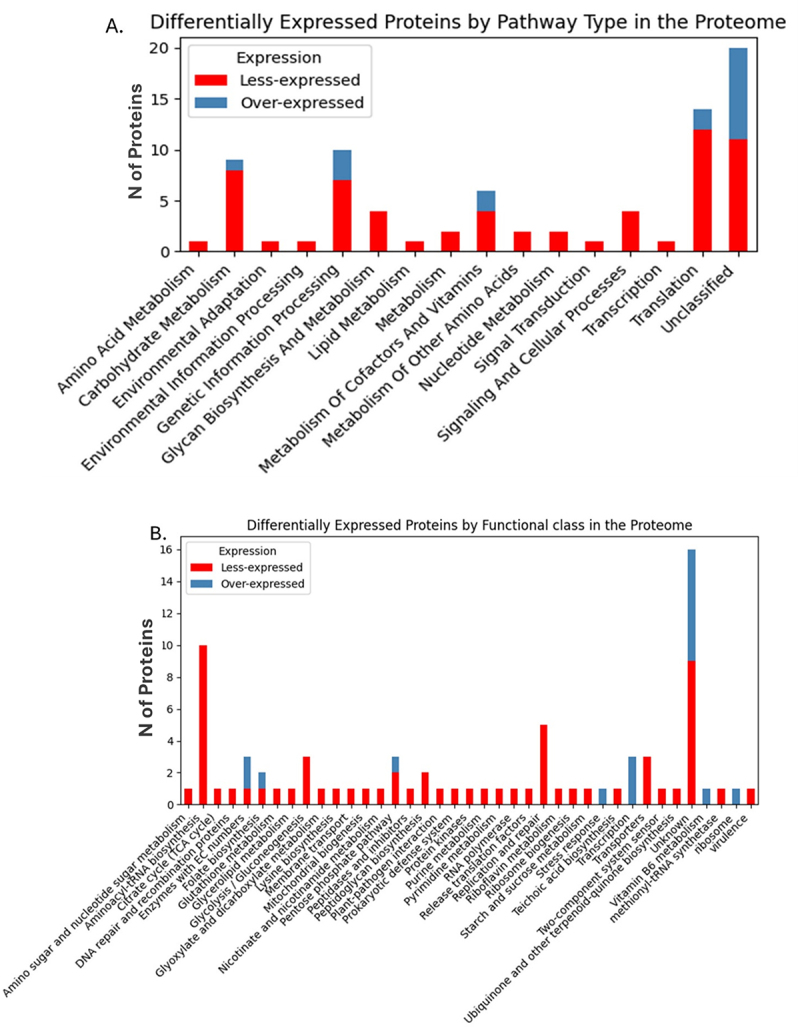
Classification of differentially abundant proteins in the proteome of *S. aureus* exposed to Adh2. (A) Pathway-based classification. (B) Functional class-based classification. Less-produced proteins are indicated in red) and over-produced in blue. Each condition was performed in biological triplicate. Differential protein expression was considered significant with Fold change ≥ 1.5 or ≤–1.5, and a *p* < 0.05. Functional analysis for the identified proteins was obtained using the BLAST Koala tool (https://www.Kegg.jp/blastkoala/). The corresponding KEGG pathways and functional categories were then assigned using the KEGG Mapper.

As shown in Figure 5(A), most differentially expressed proteins were involved in essential metabolic pathways, including amino acid, carbohydrate, glycan, lipid, cofactor/vitamin, and nucleotide metabolism. These proteins were predominantly downregulated, indicating a global suppression of metabolic activity under Adh2 exposure. Additional downregulated proteins were associated with cellular signaling, membrane transport, and genetic regulation (DNA replication, transcription and aminoacyl-tRNA biosynthesis) (Figure 5(B)), suggesting impaired nutrient uptake, nucleic acid maintenance, and protein synthesis. Iron acquisition systems were also repressed, and signal transduction pathways were altered. Notably, AgrC, the histidine kinase receptor of the Agr system, was downregulated (log2FC = −0.50, *p* = 0.03), consistent with suppression of quorum sensing and virulence coordination. CvfB, an RNA-binding virulence regulator, was also downregulated (log2FC = −0.16, *p* = 0.003), reinforcing the observed attenuation of virulence. Conversely, three regulatory proteins were significantly upregulated: IcaR, a repressor of biofilm formation (log2FC = 0.72, *p* = 0.03), an anti-sigma factor (log2FC = 0.38, *p* = 0.01), and a phage anti-repressor (log2FC = 0.42, *p* = 0.02). Ygs, a post-transcriptional regulator with an S1 domain, was also upregulated (log2FC = 0.54, *p* = 0.02), and classified under stress response.

**Figure 6.**
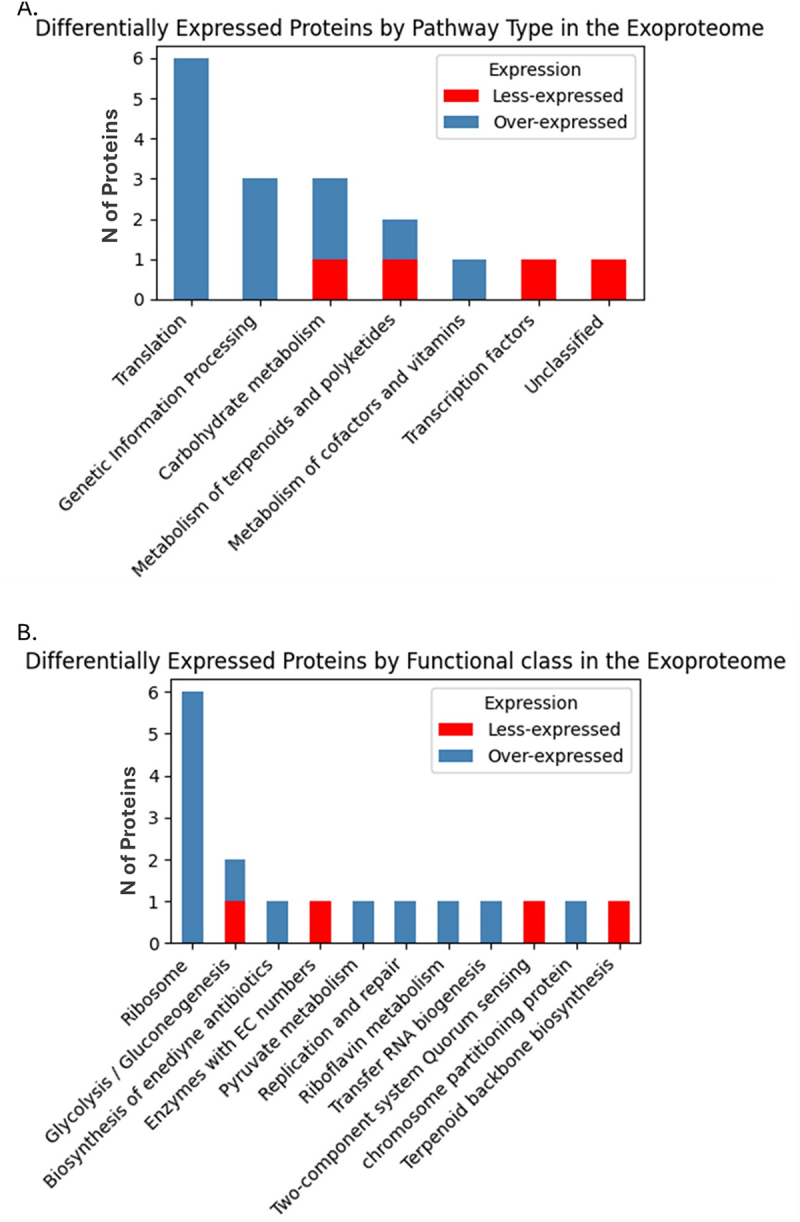
Classification of differentially abundant proteins in the exoproteome of *S. aureus* exposed to Adh2. A) Pathway-based classification. (B) Functional class-based classification. Less-produced proteins are indicated in red) and over-produced in blue. Each condition was performed in biological triplicate. Differential exoprotein expression was considered significant with Fold change ≥ 1.5 or ≤–1.5, and a *p* < 0.05. Functional analysis for the identified proteins was obtained using the BLAST Koala tool (https://www.Kegg.jp/blastkoala/). The corresponding KEGG pathways and functional categories were then assigned using the KEGG Mapper.

In the exoproteome, most of the 17 differentially abundant proteins (Table S7) were involved in metabolic pathways and were more prevalent in Adh2-treated samples, suggesting secretion via non-classical routes (Figure 6(A,B)). Signal peptide analysis revealed that only one protein (XGU33256.1) carried a classical secretion signal (Table S5). AgrA was detected in both conditions but showed reduced abundance with Adh2 (log2FC = −0.39, *p* = 0.04), suggesting altered extracellular dynamics, potentially due to changes in protein release or stability rather than classical secretion mechanisms.

### Adh2 does not affect S. aureus growth

**Figure 7.**
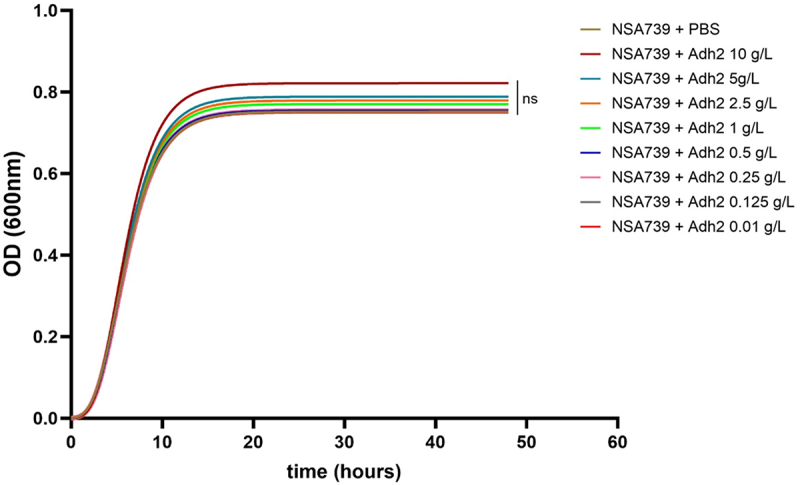
Growth curves of *S. aureus* NSA739 measured in the presence of Adh2, ranging from 0.01 g/L to 10 g/L. Growth dynamics were unaffected across all tested concentrations. Data represents mean of three biological replicates. Statistics were performed using unpaired t-test using GraphPad Prism version 10 comparint NSA739+Adh2 vs NSA739 alone (ns: not significant).

To determine whether Adh2 affects bacterial growth, NSA739 cultures were monitored over 48 h across a range of Adh2 concentrations (0.01 to 10 g/L) using a TECAN plate reader. Growth curves revealed no significant differences between Adh2-treated and control cultures. All concentrations produced growth profiles nearly identical to controls (Figure 7). These results indicate that Adh2 does not impair *S. aureus* growth.

### Adh2 promotes biofilm formation in S. aureus

**Figure 8.**
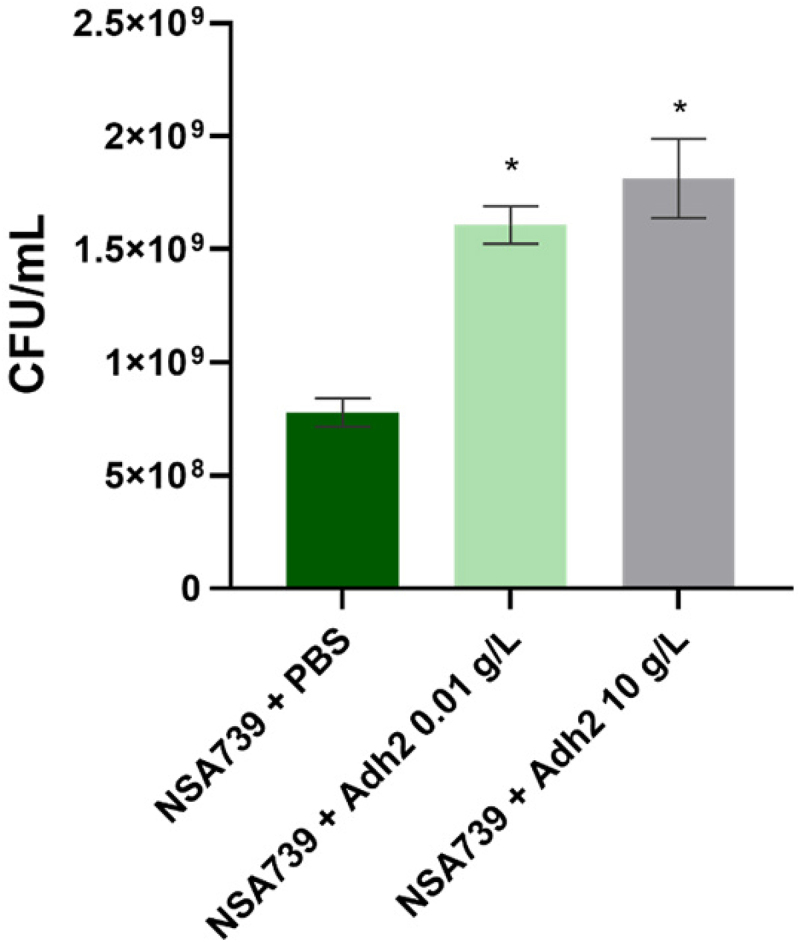
Quantification of viable cells in biofilms formed by *S. aureus* NSA739. the presence of Adh2 at low (0.01 g/L) or high (10 g/L) concentration significantly increased biofilm biomass produced after 24 h of incubation. Bars represent the mean ± standard deviation from three independent experiments, each with duplicates. Statistical analysis was performed using unpaired t-test on GraphPad prism version 10 (* *p* < 0.05, ** *p* < 0.01).

To evaluate the impact of Agr inhibition on biofilm formation, NSA739 cultures were treated with Adh2 (0.01 g/L or 10 g/L) and incubated under static conditions for 24 h. Biofilm-associated viable cells were quantified using a Quantum Cell Counter, providing CFU/mL values as a proxy for biofilm biomass. Both Adh2 concentrations significantly increased biofilm-associated CFUs compared to PBS-treated controls, with the highest effect observed at 10 g/L (Figure 8). This enhanced biofilm formation is consistent with Agr system repression, which is known to promote biofilm development. These findings suggest that Adh2 shifts *S. aureus* toward a less virulent but more persistent phenotype.

### Adh2 reduces S. aureus virulence in a zebrafish embryo Model

**Figure 9.**
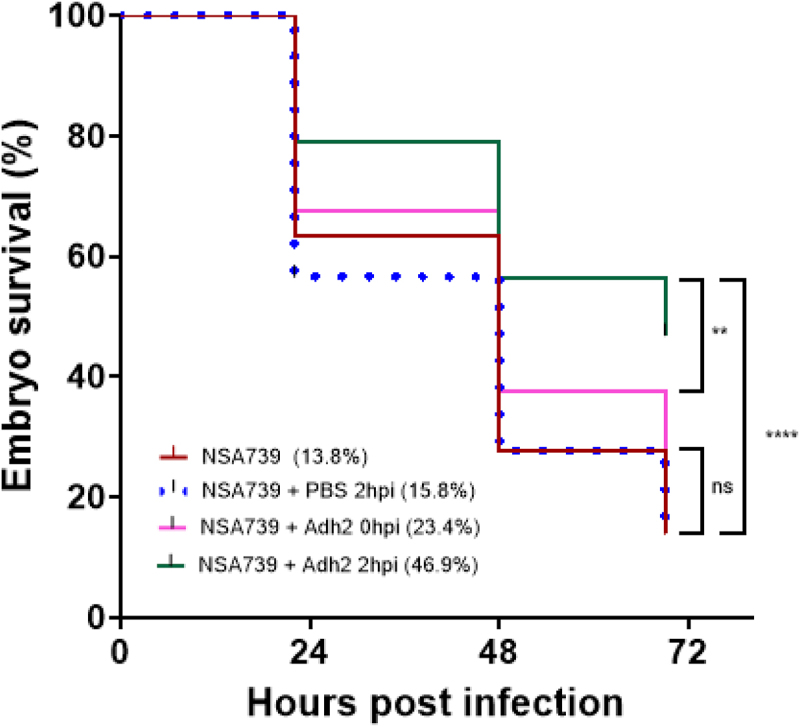
Survival curves of zebrafish embryos (Tg_mpx:umn) infected with *S. aureus* NSA739 and treated with Adh2 (10 g/L). Embryos (*n* = 35 per group) were injected with bacteria alone, co-injected with Adh2 (0-h post-injection, hpi), or treated with Adh2 (2 hpi), PBS-treated embryos served as controls. Survival was monitored over 72 h. Data represents three independent biological replicates. Statistical analysis was performed using the Log-rank (Mantel–Cox) test; (****p < 0.0001). Survival percentages at 72 hpi are indicated.

To validate the anti-virulence effect of Adh2 *in vivo*, zebrafish embryos were infected with NSA739-GFP and treated with Adh2 (10 g/L) either at the time of infection (0 hpi) or 2 hpi by intravenous injection. This concentration was selected based on previous *in vitro* results demonstrating consistent inhibition of the Agr system and virulence gene repression. In parallel, two control groups were included (infection alone and PBS injection at 2 h after injection with *S. aureus*). Survival was monitored over 72 h. As shown in Figure 9, embryos infected with NSA739 alone exhibited high mortality, with only 13.8% survival at 72 hpi, similar to PBS-treated controls (15.8%). Adh2 co-injection modestly improved survival (23.4%, *p* = ns), while delayed administration at 2 hpi significantly increased survival to 46.9% (*p* < 0.0001 vs untreated). Analysis of bacterial load showed that embryos injected with Adh2 2 h after infection had significantly reduced bacterial load at 24 hpi, while this was not the case when Adh2 was injected simultaneously with the bacteria (figure S3). These results confirm that Adh2 reduces *S. aureus* virulence *in vivo*, even when administered post-infection, and supports its potential to alter the capacity of this bacterium to cause rapidly fatal systemic infection.

## Discussion

Understanding the molecular interplay between *S. aureus* and other bacteria in polymicrobial environments such as chronic wounds is crucial for developing alternative therapeutic strategies. In this study, we demonstrate that Adh2, a secreted protein from *H. kunzii*, profoundly reprograms *S. aureus* physiology by repressing the Agr quorum sensing system, downregulating metabolic activity, and enhancing adhesion. These effects were confirmed through RT-qPCR, RNA-Seq, proteomic analyses, and validated *in vivo* using a zebrafish infection model, where Adh2 administration significantly reduced *S. aureus*-induced mortality.

The observed repression of *agrA* and *hla*, alongside upregulation of *spa*, is consistent with the established role of Agr in virulence regulation activating toxins while repressing surface adhesins [7]. These transcriptional changes were consistent across both early (1 h) and late (24 h) timepoints and at two Adh2 concentrations. The lower dose (0.01 g/L) mirrors concentrations used in the previous quorum sensing inhibitor studies [36,37], while the higher dose (10 g/L) corresponds to the maximal nontoxic concentration validated in zebrafish [38]. Importantly, deletion of the Adh2 region encompassing the conserved CDFIM motif abolished the regulatory effects observed with the wild-type protein, indicating that this region is required for Adh2 activity. However, because the deleted segment comprises approximately 60 amino acids in addition to the CDFIM pentapeptide, the present data does not allow the observed phenotype to be attributed specifically to the CDFIM sequence alone. Other residues within the deleted region may also contribute to the activity of Adh2. Nevertheless, the results support the involvement of this region in Agr modulation and are consistent with the hypothesis that Adh2 may function as a mimic of endogenous autoinducing peptides and interfere with Agr signaling [9,19]. This is particularly relevant, as recent studies have highlighted the potential of synthetic or natural AIP analogues to modulate Agr activity and repress virulence [39,40]. While our data indicate that Adh2 affects the Agr system, the precise mechanism of action remains to be determined. In particular, whether Adh2 directly interacts with the AgrC sensor kinase or indirectly modulates quorum sensing signaling cannot be concluded from the present study. Future work will aim to characterize potential interactions between Adh2 and components of the Agr system, as well as to evaluate its effects in agr-deficient strains. To explore the broader regulatory effects of Adh2, we performed transcriptomic and proteomic profiling under early exposure conditions (0.01 g/L, 1 h). This setup, designed to focus on specific Agr-related responses, follows anti-virulence approaches that use early host–pathogen interaction snapshots to identify actionable targets [41]. RNA-Seq data revealed strong repression of virulence regulators, particularly the *agrA–D* operon, following Adh2 exposure. Beyond Agr, other regulatory pathways were also affected. Notably, the two-component system SrrAB, which controls gene expression under hypoxic and oxidative stress, was significantly downregulated. This system regulates fermentative metabolism, toxin production, and biofilm development under host-like, low-oxygen conditions [42,43]. Its repression suggests that Adh2 interferes with bacterial adaptation to host environments, thereby attenuating virulence in such niches. Genes encoding accessory secretion system components (*asp2* and *asp3*), associated with the SecA2 pathway and the export/glycosylation of virulence-related proteins, were also repressed. Although the precise function of this system in *S. aureus* remains unclear, previous studies have linked it to the secretion of glycosylated surface proteins involved in immune modulation and adhesion [44,45].

Several immune evasion genes were also repressed, including *cap8M* (capsule biosynthesis, essential for resistance to phagocytosis and complement deposition [46]), *atl* (autolysins, critical for cell separation, peptidoglycan remodeling [47]), *ausA*/*B* (aureusimines biosynthesis, small peptide toxins implicated in immune modulation and bacterial fitness [48]) and *sbi* (staphylococcal binder of immunoglobulin, which disrupts complement activation and Fc-mediated phagocytosis by binding IgG and factor H [49]). These changes indicate reduced resistance to host defenses and support an anti-virulence effect of Adh2. Additionally, a gene encoding a phage portal protein was repressed, suggesting interference with prophage mobilization, a mechanism often linked to virulence gene dissemination [50]. Conversely, genes associated with adhesion and colonization were upregulated. These include *vWf* (von Willebrand factor, promoting attachment to extracellular matrix components [51,52]), *fnbpB* (fibronectin-binding protein B, involved in tissue colonization and biofilm formation [52,53]), *coa* (staphylocoagulase, which activates host prothrombin to form protective clots [54]), *lrgA* (part of an antiholin-like system linking cell wall remodeling to biofilm structuring [55]), and *ftsL* (septum formation and cell division [56]). Upregulation of *sarS*, a regulator known to inhibit *agr* while enhancing adhesin expression [57,58], reinforces the observed adhesion-oriented phenotype. Finally, *srtA* (sortase A), responsible for anchoring surface proteins to the peptidoglycan [59], further supports a colonization strategy. This shift toward a sessile, adhesive phenotype reflects a transition from acute virulence to persistence, consistent with Agr inhibition.

Transcriptomic data also revealed broad suppression of central metabolic pathways, a phenomenon increasingly recognized as a strategy to attenuate *S. aureus* virulence [60]. For example, Rudra and Boyd demonstrated that disruptions in central carbon metabolism broadly alter virulence factor expression and attenuate pathogenicity [61]. Similarly, Stephens *et al*. showed that metabolic regulators such as CcpA and CodY, which control carbon and amino acid metabolism, indirectly modulate *agr* expression and toxin production [62]. The repression of metabolic pathways observed here likely contributes to the global anti-virulence effect exerted by Adh2. Several of the differentially expressed genes identified in this study are known to be regulated, directly or indirectly, by the Agr quorum sensing system. For instance, the downregulation of *cap8M*, which is positively regulated by Agr, is consistent with reduced Agr activity. Similarly, decreased expression of virulence-associated genes such as *atl, ausA, ausB*, and *sbi* further supports a global repression of Agr-dependent functions. In addition, the upregulation of adhesion-related genes such as *fnbB* and *vwb*, which are typically repressed by Agr, is consistent with a shift toward a more adhesive, less invasive phenotype, characteristic of Agr inhibition. The modulation of *srrB*, part of the SrrAB two-component system known to interact with Agr signaling, may reflect secondary regulatory effects and cross-talk between global regulatory networks. These findings indicate that Adh2 treatment induces a transcriptional profile consistent with Agr suppression, while also affecting additional regulatory pathways. Further studies using agr-deficient mutants will be required to distinguish direct from indirect effects. Together, these transcriptomic changes highlight a global reprogramming of *S. aureus* toward reduced virulence, altered metabolism, and enhanced colonization capacity under Adh2 exposure.

Proteomic analysis confirmed the transcriptomic findings, with downregulation of proteins involved in metabolism, membrane transport, iron acquisition, DNA replication, RNA polymerase activity, and aminoacyl-tRNA biosynthesis. Notably, AgrC abundance was reduced, reinforcing the disruption of quorum sensing. These observations are consistent with those of Durand *et al*., who reported that *S. aureus* virulence was impaired during coculture with *H. kunzii*, with repression of iron-related pathways, changes in cell wall structure, and reduced metabolic activity [21]. Collectively, these results highlight how interference with regulatory and metabolic networks, either through interspecies interactions or direct inhibition by agents like Adh2 can weaken *S. aureus* virulence.

Despite widespread repression, some regulatory proteins were upregulated. The anti-sigma factor, typically activated under cell envelope stress or during stationary phase, suggests Adh2-mediated modulation of global transcriptional responses [63]. Upregulation of a phage anti-repressor supports suppression of prophage activation, a process often associated with virulence gene mobilization [64], further limiting auxiliary virulence mechanisms commonly triggered during infection. In addition, the downregulation of AgrC at the protein level, together with the decrease in CvfB, an RNA-binding regulator involved in virulence control, further supports attenuation of Agr-associated virulence pathways. Conversely, the upregulation of IcaR, a repressor of biofilm formation, is consistent with a shift in the balance between virulence and biofilm-associated phenotypes, which is known to be influenced by Agr activity. Altogether, these observations indicate that Adh2 not only inhibits the Agr quorum sensing system but also induces broader regulatory adjustments, affecting interconnected networks involved in virulence, stress response, and biofilm dynamics. These proteomic changes are consistent with the transcriptional profile observed by RNA-seq, supporting a global shift toward reduced virulence and altered regulatory activity under Adh2 treatment. The downregulation of AgrC at the protein level further confirms inhibition of the Agr system and is in agreement with transcriptomic data, indicating a coordinated multi-omics response.

Exoproteomic analysis revealed increased abundance of metabolics proteins in Adh2-treated samples, likely secreted via non-classical secretion mechanisms. However, the predominance of cytoplasmic proteins in the exoproteome suggests that a significant fraction of detected proteins may originate from passive release or cell lysis rather than active secretion. In addition, although cultures were diluted into fresh medium prior to Adh2 treatment, proteins secreted during the growth phase preceding treatment may have accumulated in the extracellular environment. This potential accumulation, combined with limited protein turnover, may have masked changes in secretion dynamics. This could explain the absence of a more pronounced decrease in secreted virulence factors, such as toxins typically regulated by the Agr system. In *S. aureus*, such non-canonical secretion has been attributed to membrane vesicles, which can carry cytoplasmic proteins, including Ldh and GyrB, into the extracellular environment [65,66]. This process is thought to serve as a disposal route for proteins no longer needed by the cell, as previously shown for glycolytic enzymes like aldolase and enolase [67], and more generally supported by the role of PSMα peptides in promoting nonspecific excretion of cytoplasmic proteins [68]. AgrA was also detected extracellularly, both in the presence and absence of Adh2, supporting the hypothesis that it may be released through non-classical secretion pathways, similarly to metabolic proteins. However, its abundance was reduced under Adh2 exposure. Importantly, bacterial response regulators like AgrA exist in both phosphorylated and non-phosphorylated forms. Prior studies support the existence of an intracellular pool of unphosphorylated AgrA [69,70], and the well-described non-classical export of cytoplasmic proteins in *S. aureus* provides a plausible route for the extracellular detection of unused AgrA, although direct evidence for secretion of the unphosphorylated form remains lacking. Previous proteomic studies have also reported the presence of intracellular regulatory proteins such as SarA, MgrA, and Rot, in exoproteome fractions, potentially due to vesicle-mediated export or sequestration [66].

Phenotypically, Adh2 did not affect *S. aureus* growth across tested concentrations, consistent with an anti-virulence effect rather than a bacteriostatic or bactericidal action. Anti-virulence agents targeting quorum sensing or global regulators typically attenuate toxin production and virulence behaviors without affecting growth rate, as reviewed for *S. aureus* [71]. For example, Sully *et al*. showed that selective inhibition of the Agr system by the savarin molecule reduces RNAIII and virulence factor expression while leaving exponential-phase growth unchanged, illustrating that Agr repression can decouple pathogenicity from replication [17].

Adh2 significantly increases biofilm formation, a known consequence of Agr inhibition. The Agr system is a key negative regulator of biofilm formation in *S. aureus*, when *agr* is repressed, the bacterium tends to adopt a sessile, adhesive lifestyle rather than producing toxins and dispersing [72]. Several studies have shown that *agr*-defective or *agr*-suppressed strains exhibit increased biofilm formation and reduced acute virulence, supporting our findings [73,74].

Finally, *in vivo* administration of Adh2 at 10 g/L significantly improved survival in zebrafish embryos infected with *S. aureus* (46.9% vs 13.8% in controls), particularly when administered 2 hpi. In contrast, co-administration at 0 hpi resulted in a more limited protective effect. This difference may reflect the importance of initial host–pathogen interactions in establishing infection dynamics, which could be prematurely disrupted by immediate Adh2 exposure. Delayed administration likely allows early bacterial signaling and colonization processes to occur before Adh2 interferes with Agr-mediated virulence regulation, resulting in a more pronounced attenuation of pathogenicity. These results align with previous studies validating anti-virulence compounds in zebrafish models. The zebrafish infection model has been effectively used to evaluate anti-virulence strategies targeting bacterial regulatory systems, including Agr [38]. For instance, Fries *et al*. demonstrated the efficacy of sorangicin A in zebrafish larvae infected with *S. aureus*, highlighting the model’s utility for early-stage therapeutic validation [38]. Moreover, targeting specific bacterial enzymes such as the ATPase ClpP with ZG197 has proven effective in zebrafish and mouse models, underscoring the translational value of this approach [75]. Treatment with Adh2 resulted in a significantly lower bacterial burden at 24 hpi relative to untreated infected embryos, suggesting reduced virulence factor expression prevented uncontrolled replication of *S. aureus*, but further experiments are required to define the precise mechanisms. Future studies should define the minimal effective dose and therapeutic window of Adh2, explore combination therapies with anti-biofilm agents, and investigate targeted delivery systems for chronic wound applications. Combining Adh2 with conventional antibiotics may further enhance clinical outcomes and reduce resistance selection, an approach increasingly advocated in combating multidrug-resistant pathogens [71].

In conclusion, we characterized the anti-virulence activity of Adh2, a secreted AIP-like protein from *H. kunzii*, and demonstrated its ability to inhibit the *S. aureus* Agr quorum sensing system. Through integrated transcriptional, proteomic, phenotypic, and *in vivo* analyses, we showed that Adh2 shifts *S. aureus* toward a less virulent, metabolically suppressed, and colonizing-oriented state without impairing bacterial growth. These findings highlight the therapeutic potential of Adh2 as a targeted anti-virulence agent and support the broader concept of quorum sensing interference as a strategy to combat bacterial pathogenesis. Commensal-derived proteins such as Adh2 may offer promising avenues for managing chronic and antibiotic-resistant infections.

## Supplementary Material

Supplementary tablesR.xlsx

Supplementary DataR.docx

## Data Availability

All raw data related to the heart experiments, qPCR analyses, biofilm assays, and zebrafish studies are available on Figshare at DAHER, Riham (2025). Raw GraphPad Prism data for the paper A secreted AIP-Like peptide from *Helcococcus kunzii* inhibits the Agr Quorum Sensing System of *Staphylococcus aureus*. Figshare. Dataset. https://doi.org/10.6084/m9.figshare.30598655 [76]. Supplementary data, tables and additional figures are also provided in the same Figshare repository with the same doi. The RNA-Seq raw data generated in this study have been deposited in the NCBI GenBank database under BioProject accession number PRJNA1314433. The dataset includes six BioSamples: SAMN50933932 (R1_NoProt), SAMN50933933 (R2_NoProt), SAMN50933934 (R3_NoProt), SAMN50933935 (R1_Prot), SAMN50933936 (R2_Prot), and SAMN50933937 (R3_Prot). The mass spectrometry proteomics data have been deposited to the ProteomeXchange Consortium via the PRIDE [77] partner repository with the dataset identifier PXD069181 and 10.6019/PXD069181.
