# Supplementary material for: A secreted AIP-Like peptide from Helcococcus kunzii inhibits the Agr quorum sensing system of Staphylococcus aureus
Source: Virulence. 2026 Jul 3;17(1):2692776. doi: 10.1080/21505594.2026.2692776 (PMC13336275; doi:10.1080/21505594.2026.2692776)
Supplement: Supplementary DataR.docx [file KVIR_A_2692776_SM1451.docx]

**Supplementary Figures**


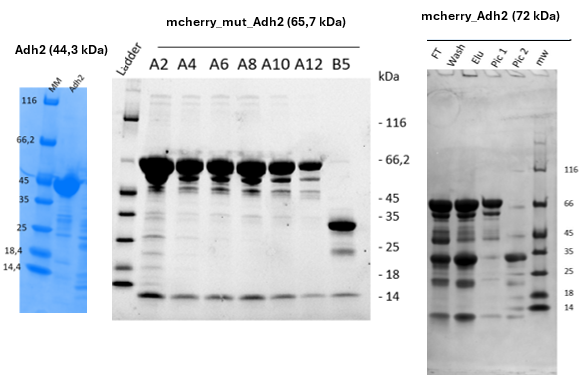


**Figure S1: SDS-PAGE analysis of purified Adh2, mcherry_mut_Adh2 and mcherry_Adh2 proteins.** Recombinant proteins were produced in *E. coli* BL21(DE3) and purified by nickel chelate affinity chromatography. All proteins were analyzed under denaturing conditions using a 4-20% SDS-PAGE gel and stained with Coomassie Brilliant Blue. Bands correspond to expected sizes, confirming successful production and purification of target constructs.


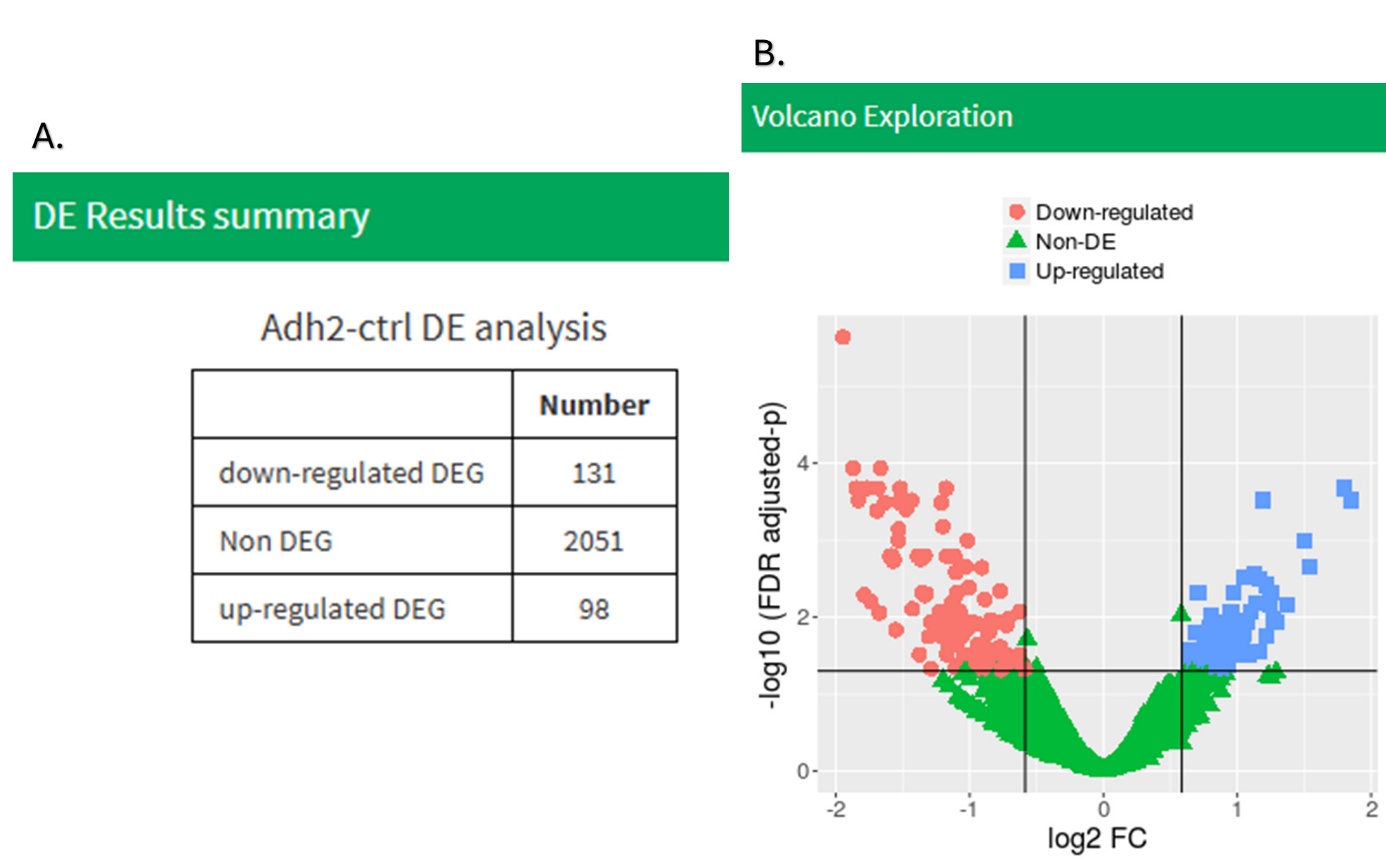


**Figure S2: Overview of global differential genes expression in S. aureus following Adh2 exposure.** (A) Summary of differentially expressed genes (DEGs) identified by DESeq2 comparing Adh2-treated and untreated S. aureus NSA739. (B) Volcano plot of all analyzed genes showing log2 fold change (X-axis) versus –log10 adjusted p-value (Y-axis). P-values were adjusted using the false discovery rate (FDR = 0.05) correction. Downregulated genes (red), upregulated genes (blue), and non-significant genes (green) are distinguished by fold change and statistical thresholds.


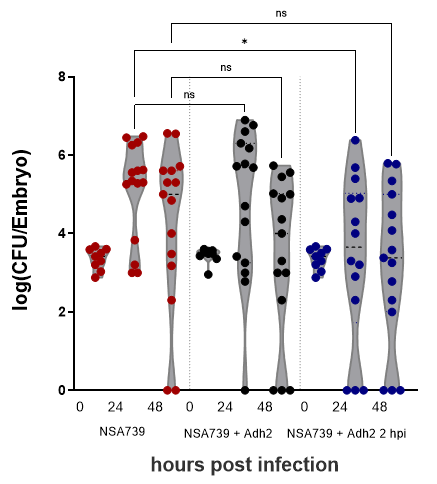


**Figure S3:** **Bacterial load (CFU) in zebrafish embryos infected with *S. aureus* NSA739 and treated with Adh2.** Violin plots showing the distribution of bacterial counts (log (CFU/embryo)) at 0, 24, and 48 hours post infection (hpi). Embryos were injected with *S. aureus* NSA739 alone or co-injected with Adh2 (NSA739 + Adh2; average 2955 CFU/embryo). The embryos injected with NSA739 alone were randomized and 50% were treated with Adh2 2 hours post-infection (NSA739 + Adh2 2 hpi). CFU data are represented in violin plots, with scattered data points. Data were normally distributed with an D’Agostino & Pearson test. Significance was determined using One-way Anova (Sidaks’ multiple comparison test). * P= 0.031. ns = non-significant.


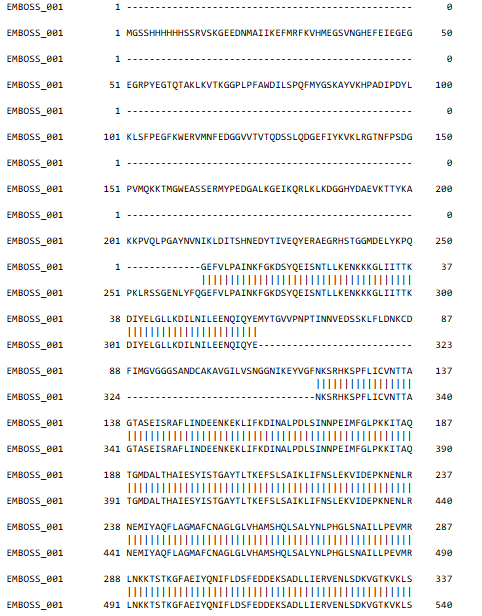


B

A

**Figure S4:** **Pairwise sequence alignment of A) wild-type Adh2 and the B) mch_mut_Adh2 mutant, showing deletion of the CDFIM motif and adjacent residues, generated using the EMBL-EBI Job Dispatcher sequence analysis tools (2024).**


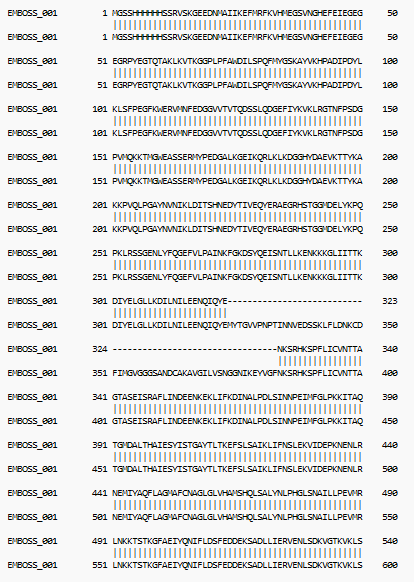


B

A

**Figure S5:** **Pairwise sequence alignment of A) mCherry-Adh2 and B) mCherry-mutant Adh2 showing the deletion of the conserved CDFIM motif and adjacent amino acids, generated using the EMBL-EBI Job Dispatcher sequence analysis tools (2024).** Vertical bars indicate conserved residues between the two sequences. The deleted region in the mutant construct includes the conserved CDFIM motif and surrounding amino acids, while the mCherry fusion remains identical in both constructs.
